# Anti-Inflammatory Effects of an Extract of *Polygonum hydropiper* Stalks on 2,4,6-Trinitrobenzenesulphonic Acid-Induced Intestinal Inflammation in Rats by Inhibiting the NF-*κ*B Pathway

**DOI:** 10.1155/2018/6029135

**Published:** 2018-05-07

**Authors:** Wei Zhang, Yingni Pan, Shouhe Qu, Dongmei Wang, Song Cheng, Xiaoqiu Liu

**Affiliations:** ^1^School of Traditional Chinese Materia Medica, Shenyang Pharmaceutical University, 103 Wenhua Road, Shenyang, Liaoning 110016, China; ^2^School of Pharmacy, Shenyang Pharmaceutical University, 103 Wenhua Road, Shenyang, Liaoning 110016, China

## Abstract

The stalks of *Polygonum hydropiper* L. *(PHL)* have been traditionally used in clinical practice for thousands of years in China to treat various inflammatory diseases. However, little research has been conducted to investigate the anti-inflammatory effects of *PHL* on TNBS-induced intestinal inflammation in rats. The aim of the present study was to investigate the anti-inflammatory effects and to explain the underlying mechanism of *PHL* on TNBS-induced intestinal inflammation in rats. *PHL* (125, 250, and 500 mg/kg) was given for 7 consecutive days to rats with intestinal inflammation induced by TNBS. Oral administration of an aqueous extract of a high dose of *PHL* (H-*PHL*) significantly improved TNBS-induced symptoms such as the macroscopic score and histological examination. H-*PHL* treatment significantly ameliorated the activity of MPO and improved the GSH content. In addition, there was a downregulation of the TNBS-induced increase in the activity of iNOS and levels of Cox-2, TNF-*α*, and IL-1*β* while the protein expression of NF-*κ*B was significantly unregulated after administration of H-*PHL*. The present findings suggested that H-*PHL* has a protective effect on experimental intestinal inflammation in rats and its anti-inflammatory effects are closely related to inhibition of NF-*κ*B signal pathways.

## 1. Introduction

Inflammatory bowel disease (IBD) is a chronic digestive tract disease, mainly Crohn's disease and ulcerative colitis (UC), which is characterized by continuous or recurrent diffuse inflammation of the colon mucosa, and submucosa, and its clinical manifestations are diarrhea, blood in the stool, and abdominal pain [1, 2]. To date, the abnormal exacerbated immune response and overexpression of proinflammatory mediators are recognized as major risk factors for the development of IBD. Currently, the main therapeutic strategy for human IBD is to interfere with the multiple stages of the inflammatory cascade to downregulate the exacerbated immune response by treatment with anti-inflammatory medication (5-aminosalicylic acid and glucocorticosteroids) and immunomodulators (azathioprine, mercaptopurines, and cyclosporine) [3, 4]. Several investigations have demonstrated the efficacy of these treatments which have made a substantial contribution to the treatment of the disease. However, these drugs have many serious side effects and there is a high recurrence of IBD, and no definitive therapies are available for this disorder [5, 6]. Therefore, seeking assistance from complementary and alternative therapies is of great interest. Chinese herbal medicine, an important category of complementary and alternative medicine, has been proven to be an efficacious and safe treatment option for patients with IBD [7–9]. The stalks of *Polygonum hydropiper* L. *(PHL)*, commonly used as a traditional Chinese medicine, have been widely used in the treatment of inflammatory diseases. The compounds present in *PHL* have been reported to have multiple effects and involve antifungal agents (polygodial and warburganal), anti-inflammatory agents (polygonolide), and antioxidants (hydropiperoside, rhamnazin, persicarin) [10–14]. The *PHL* agents extracted with different solvents, such as methanol, hexane, and ethyl acetate, all exhibited significant activity on acetic acid-induced writhing [15]. In addition, the *PHL* methanol extract has a potential anti-inflammatory effect [16]. *PHL* aqueous extract has been widely used as a herbal medicine; however, its anti-inflammatory effects on IBD have not been studied until now. In addition, the mechanism involved in the protective effect against intestinal inflammation remains unknown.

Accordingly, in the present study, we examined the protective effects of a *PHL* aqueous extract on TNBS-induced intestinal inflammation using a macroscopic score and histological analysis as well as by determination of the MPO activity and GSH content. In order to investigate the underlying mechanisms of a water decoction of *PHL* in treating inflammatory injury in experimental intestinal inflammation, the production of proinflammatory cytokines, such as the production of TNF-*α*, IL-1*β*, and Cox-2; the activity of iNOS; and activation of the NF-*κ*B signaling pathway were also evaluated by enzyme-linked immunosorbent assay (ELISA) and Western blotting.

## 2. Materials and Methods

### 2.1. Chemicals and Reagents

TNBS was obtained from Sigma Chemical Co. Ltd. (St. Louis, MO, USA). Sulphasalazine (SAZ) was obtained from Sine Pharmaceutical Co. Ltd. (Shanghai, China). Sodium carboxymethyl cellulose (CMC-Na) was obtained from Sanchine Pharmaceutical Co. Ltd. (Harbin, China). The kits for biochemical analysis of MPO, GSH, LDH, iNOS, Cox-2, TNF-*α*, and IL-1*β* were obtained from Nanjing Jiancheng Bioengineering Institute (Nanjing, China).

### 2.2. Preparation of Aqueous Extract of *Polygonum hydropiper* L*. (PHL)*

Stalks of *Polygonum hydropiper* L. were obtained from Hainan Huigu Pharmaceutical Company (Hainan, China) and authenticated by Dr. Xiaoqiu Liu of Shenyang Pharmaceutical University, and a voucher specimen was deposited in the School of Traditional Chinese Materia Medica of Shenyang Pharmaceutical University. Then, 30 g of stalks of *PHL* was immersed in 360 mL distilled water for 30 min and then extracted at 100°C for 1.5 h. The supernatant was collected by filtering, and the residue was mixed with 300 mL distilled water and boiled again for another 60 min. The supernatants were combined, filtered, and concentrated to a volume equivalent to 3 g/mL plant material. The concentrated solution was stored at 4°C.

### 2.3. Animals

Specific pathogen-free- (SPF-) grade male Sprague Dawley (SD) rats (200 ± 20 g) were obtained from the Experimental Animal Center of Shenyang Pharmaceutical University and maintained at a temperature of 22–24°C and a relative humidity of 45–65%. The rats were housed in polypropylene cages with ad libitum access to food and water in a room with a 12 : 12 light/dark cycle. All animal care was in accordance with the Guidelines for Animal Experimentation of Shenyang Pharmaceutical University, and the protocol was approved by the Animal Ethics Committee of the institution. All animals were acclimatized for 1 week before the experiment was started.

### 2.4. Induction of Intestinal Inflammation

Rats were randomly divided into 6 groups, a nonintestinal inflammation (NII) control group, a TNBS control group, a SAZ group, a low-dose *Polygonum hydropiper* L.(L-*PHL*) group, a medium-dose *Polygonum hydropiper* L. (M-*PHL*) group, and a high-dose *Polygonum hydropiper* L. (H-*PHL*) group (5–8 animals/group). Intestinal inflammation was induced with TNBS as previously described. Animals were fasted overnight and anesthetized by ether inhalation before induction of intestinal inflammation. The model group and each treatment group were given 10 mg TNBS dissolved in 0.25 mL 50% ethanol (*v*/*v*) via a 2 mm diameter Teflon cannula inserted 8 cm into the anus. An equal volume of 50% ethanol was given to the rats in the normal control group by intracolonic injection. During and after TNBS administration, the rats remained in a head-down position to prevent liquid outflow until they recovered from the anesthesia [17–19]. The rats were then treated with *PHL* aqueous extracts (125, 250, and 500 mg/kg) or with SAZ (120 mg/kg) as a positive control for 7 consecutive days.

### 2.5. Macroscopic Assessment

Rats were monitored daily for changes in body weights for a period of 8 days. Once the animals were killed, the abdominal cavity of each animal was opened along the middle line, the colon was drawn out and placed on an ice-cold plate, and fat and mesentery were removed. After opening longitudinally, the colon was washed with cold saline to remove fecal residues. Then the weight and length of the colon were recorded. Each colon was immediately examined visually, and the damage to the colon was assessed using scores from 0–10 by three observers who were blinded to the group, according to the criteria described by Bell et al. [20]. Representative whole gut specimens taken from a region of the inflamed colon corresponding to the segment adjacent to the gross macroscopic damage were used for the histological examination. The left material of the distal part (8 cm from the anus) divided into different pieces was stored at −80°C and subsequently used for the following biochemical assays.

### 2.6. Histological Examination

Colonic tissues were fixed in 10% formalin in phosphate-buffered saline for 24 h, dehydrated in increasing concentrations of ethanol, and subsequently embedded in paraffin. Sections of 4 *μ*m thick colon were stained with hematoxylin and eosin (H&E), and a histological damage score was obtained based on three parameters according to the criteria previously described by Stucchi et al. [21].

### 2.7. Assessment of the GSH Content and Activity of MPO and iNOS

The colon segments were homogenized in 1 mL cold saline, the homogenates were then centrifuged at 3500*g* for 15 min at 4°C, and the supernatant was used for the measurement of MPO, GSH, and iNOS according to the manufacturer's instructions (Nanjing Jiancheng Bioengineering Institute, China).

### 2.8. Determination of Cox-2, TNF-*α*, and IL-1*β* Using ELISA Kits

The production of inflammatory cytokines, TNF-*α*, IL-1*β*, and Cox-2, in the rat colon, were determined by standard enzyme-linked immunosorbent assay (ELISA) kits specific for murine cytokines according to the manufacturer's instructions (Nanjing Jiancheng Biotech Co. Ltd., China).

### 2.9. Western Blot Analysis

Colon tissues were homogenized with a handheld homogenizer on ice and centrifuged at 4°C (3500*g*, 15 min), and the supernatants were collected. The protein concentration in the colon samples was determined by BCA. Equal amounts of protein (40 *μ*g) were subjected to electrophoresis on 12% SDS-PAGE at 80–120 V for 120 min, and then the target protein at the appropriate place on the gel compared with the marker was transferred to a PVDF membrane. Blots were blocked with 5% (*w*/*v*) skimmed milk and incubated in primary antibodies. Subsequently, the blots were washed with 0.01 M Tris-buffered saline containing 0.05 Tween-20 (TBST) for 10–15 min 3 times. The blots were then incubated with horseradish peroxidase-conjugated secondary antibodies (Cell Signaling Technology Inc.) and developed by enhanced chemiluminescence (ECL) Western blotting detection reagents (Thermo Scientific, Waltham, MA). Protein expression quantification was performed by densitometric analysis of the blots.

### 2.10. Statistical Analysis

All data in this study were taken from three independent experiments and then expressed as means ± standard deviation (SD) or means ± standard error of mean (SEM). The statistical significance was analyzed using one-way analysis of variance (ANOVA) with the Statistical Package for the Social Sciences (SPSS, 19.0) software. Data as a score were evaluated by nonparametric statistical analysis, and results were analyzed using analysis of the Kruskal-Wallis test. *P* values less than 0.05 were considered statistically significant.

## 3. Results

### 3.1. A Beneficial Effect of *PHL* on TNBS-Induced Macroscopic Assessment and Weight Loss

**Figure 1:**
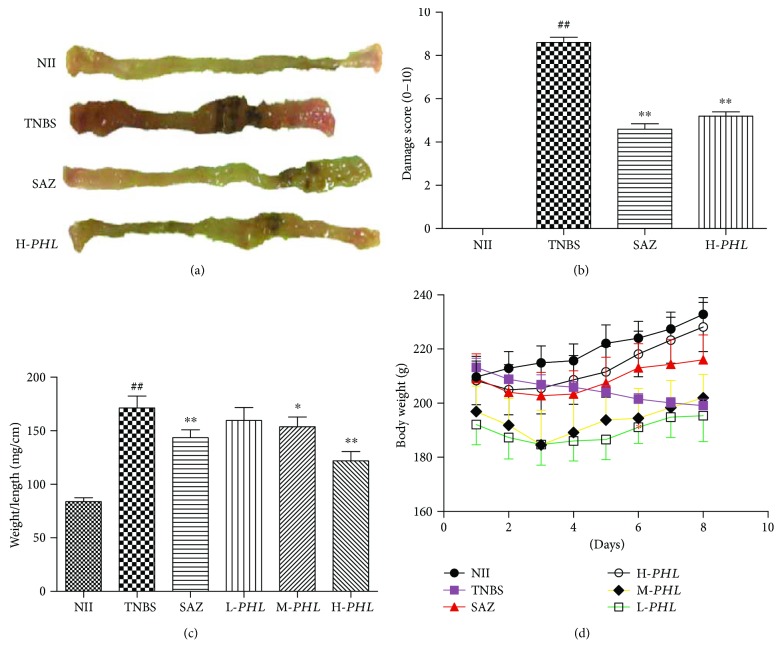
Effects of *PHL* extract on macroscopic images of the colon (a), the macroscopic damage score (b), the weight/length ratio (c), and colonic daily changes in body weight (d) in rats with TNBS experimental intestinal inflammation. Data are expressed as means ± SEM (*n* = 5); ^∗∗^*P* < 0.01 and ^∗^*P* < 0.05 versus TNBS; ^##^*P* < 0.01 versus NII.

Rats treated with TNBS showed prostration, hypomotility, diarrhea, and a significant body weight loss. By contrast, a significant reduction in body weight loss was observed in rats given H-*PHL*, on days 6, 7, and 8. Macroscopic inspection of the colon showed evidence of severe colonic mucosal damage with obvious hyperemia, bowel wall thickening, ulceration, necrosis of the mucosa, and presence of adhesions to adjacent organs. Accordingly, the TNBS control had a significantly shorter colon length compared with the nonintestinal inflammation (NII) control. Rats given oral H-*PHL* had a significantly extended colon length compared with the TNBS control. Similarly, H-*PHL* treatment significantly combatted severity of the colonic injury, reducing the macroscopic damage score, and attenuated the weight/length ratio of the rat colon (*P* < 0.01) (Figures 1(a)–1(d)).

### 3.2. Effect of *PHL* on Attenuation of Histopathological Damage in Rats with TNBS-Induced Intestinal Inflammation

**Figure 2:**
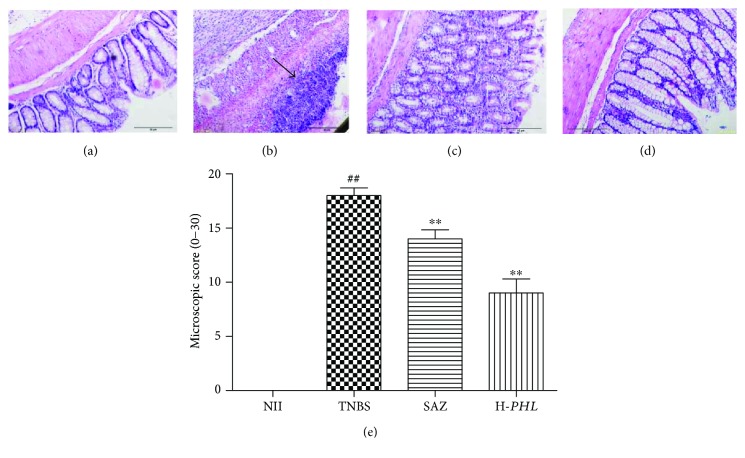
Histological sections of colonic tissue stained with hematoxylin and eosin showing the effects of different doses of *PHL* extract and SAZ in TNBS rat intestinal inflammation: (a) NII, (b) TNBS-control, (c) SAZ (120 mg/kg), (d) H-*PHL*, and (e) microscopic score. Data are expressed as means ± SEM (*n* = 5); ^∗∗^*P* < 0.01 versus TNBS; ^##^*P* < 0.01 versus NII.

Histological examination of H&E-stained colonic sections under light microscopy demonstrated that TNBS rats showed typical inflammatory changes in colonic architecture, such as the submucosa, muscularis, and serosa layers of the bowel wall. The colonic mucosa exhibited ulceration and necrosis due to destruction of the glands and epithelial cell loss. Extensive granulation tissue with the presence of a massive neutrophilic infiltration, fibroblasts, and lymphocytes was also apparent, mainly in the mucosa and submucosa (Figure 2(b)). The damage was assessed using colonic histopathological scores (range 0–30). In comparison with the NII control, TNBS rats showed significantly increased histopathological disease scores (Figure 2(e)). After treatment with H-*PHL*, the ulcer area, edema, and adhesion were significantly reduced. Similarly, H-*PHL* treatment reduced the inflammatory cells in the lamina propria. Also, some areas of the colonic mucosa structure remained intact, and the epithelium was preserved as well (Figure 2(d)).

### 3.3. Effect of *PHL* on MPO Activity and GSH Content

**Figure 3:**
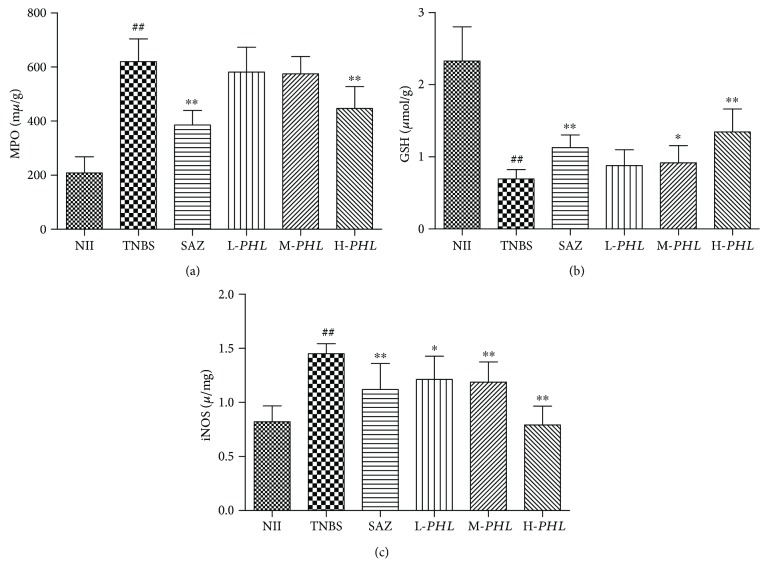
Effects of *PHL* extract (125, 250, and 500 mg/kg) and SAZ (120 mg/kg) on the colonic MPO activity (a), GSH content (b), and iNOS activity (c) in rats with TNBS-induced experimental intestinal inflammation. Data are expressed as means ± SD (*n* = 8); ^∗∗^*P* < 0.01 and ^∗^*P* < 0.05 versus TNBS; ^##^*P* < 0.01 versus NII.

The effects of the *PHL* extract on the MPO activity and GSH content in rats with TNBS-induced intestinal inflammation were examined using MPO and GSH assays. The results showed that the MPO activity in the TNBS groups, which displayed marked infiltration of inflammatory cells, has increased significantly compared with the NII rats (*P* < 0.01). The level of MPO activity was significantly reduced in the group of rats treated with H-*PHL* in relation to TNBS, as well as the SAZ group (Figure 3(a)) (*P* < 0.01). Correspondingly, the colonic glutathione content, considered as an important sign of intestinal inflammation, was reduced due to the colonic oxidative stress induced in the TNBS group by the inflammatory process [22] and was significantly increased in the group of rats treated with H-*PHL*, as well as the SAZ group (Figure 3(b)) (*P* < 0.01). These data showed an improvement in the oxidative status of rats with intestinal inflammation treated with H-*PHL* (Figure 3(b)) (*P* < 0.01).

### 3.4. Effect of *PHL* on the Activity of iNOS and the Colonic Production of Cox-2, TNF-*α*, and IL-1*β*

**Figure 4:**
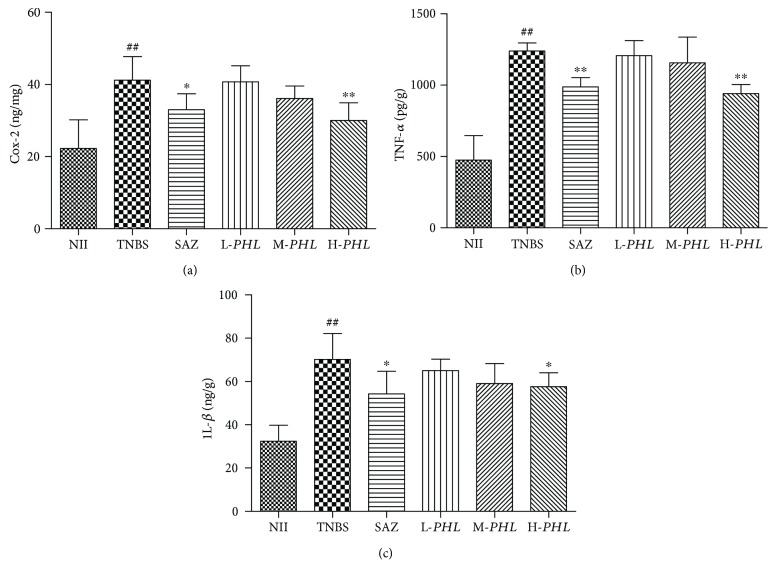
Effects of *PHL* extract (125, 250, and 500 mg/kg) and SAZ (120 mg/kg) on the colonic production of Cox-2 (a), TNF-*α* (b), and IL-1*β* (c) determined by ELISA. Data are expressed as means ± SD (*n* = 8); ^∗∗^*P* < 0.01 and ^∗^*P* < 0.05 versus TNBS; ^##^*P* < 0.01 versus NII.

The severity of colonic inflammation was also reflected by the activity of iNOS and the colonic production of Cox-2. Therefore, the effect on proinflammatory mediators, such as iNOS and Cox-2, was found in rats with TNBS-induced intestinal inflammation. The activity of iNOS and the colonic production of Cox-2 were significantly higher than the corresponding values in NII animals (Figures 3(c) and 4(a)) (*P* < 0.01). H-*PHL* treatment significantly reduced the TNBS-induced increased levels of the activity of iNOS and the colonic production of Cox-2 in rats with intestinal inflammation (Figures 3(c) and 4(a)) (*P* < 0.01).

As illustrated in Figures 4(b) and 4(c), colonic TNF-*α* and IL-1*β* levels were significantly higher in TNBS control rats than the corresponding values in NII animals (*P* < 0.01). H-*PHL* treatment significantly downregulated the TNBS-induced increase in intestinal inflammation TNF-*α* and IL-1*β* levels (*P* < 0.05 and *P* < 0.01, resp.).

### 3.5. Effect of *PHL* on NF-*κ*Bp65 Expression

**Figure 5:**
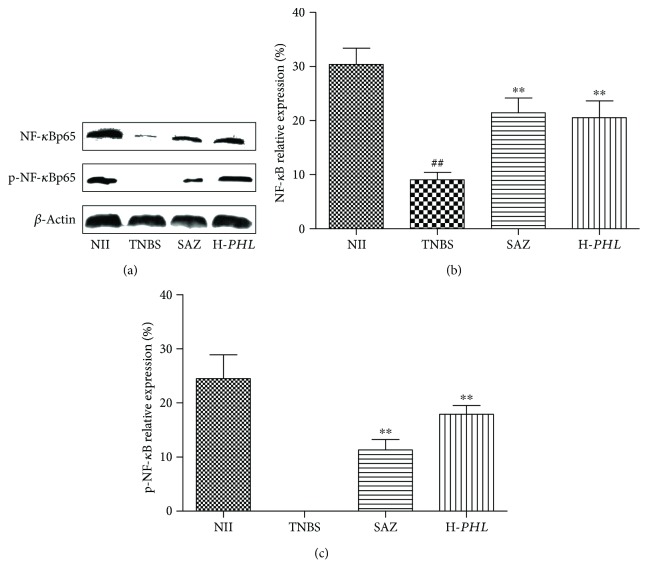
Effects of *PHL* (500 mg/kg) and sulphasalazine (SAZ) (120 mg/kg) on the colonic production of NF-*κ*Bp65 and p-NF-*κ*Bp65 determined by Western blotting. Data are expressed as means ± SD (*n* = 5); ^∗∗^*P* < 0.01 versus TNBS; ^##^*P* < 0.01 versus NII.

Immunoblotting was conducted to evaluate the expression of inflammatory mediators, NF-*κ*B and p-NF-*κ*B, in rats with TBNS-induced intestinal inflammation. As illustrated in Figure 5, TNBS-induced intestinal inflammation can significantly attenuate the levels of NF-*κ*Bp65 and p-NF-*κ*Bp65 in the cytoplasm (*P* < 0.01), while H-*PHL* treatment can significantly upregulate the protein expression of NF-*κ*Bp65 and p-NF-*κ*Bp65 in rats with TNBS-induced intestinal inflammation (*P* < 0.01), and the positive drug (SAZ 120 mg/kg) group also showed increased levels of NF-*κ*Bp65 and p-NF-*κ*Bp65 in rats (*P* < 0.01).

## 4. Discussion

It is well known that the first-line chemical and biological options widely used in the treatment of IBD have a significant effect, although their severe side effects, especially the high palindromic rate of these therapies, have caused much concern. Therefore, it was clear that we need to find some “gentle” natural drugs, with a higher efficacy but fewer side effects and a lower palindromic rate. As a traditional Chinese medicine, *PHL* has been used in the treatment of inflammatory intestinal disease in clinical situations for a long time. The present study particularly focused on the effect of *PHL* and its underlying mechanism of action on TNBS-induced intestinal inflammation, which was widely used to study the pathogenesis of colonic inflammation and to investigate potential new IBD treatments [23]. This is because intestinal inflammation induced by instillation of TNBS exhibited many similar clinical features to colonic inflammatory bowel disease [22] such as ulceration, inflammation, and leucocyte infiltration [24]. So, it was appropriate to confirm the effect of *PHL* and to identify its protective mechanism.

Using macroscopic and histological examinations, we found that H-*PHL* not only significantly reduced the wet weight of the colon and weight/length ratio but also improved the microscopic and macroscopic scores of acute colitis in model rats, parameters and indices which are used as indicators of disease-associated intestinal wall thickening and intensity of inflammation [25]. The results revealed that H-*PHL* had a beneficial effect on the intestinal inflammation damage induced by TNBS. A reduction in the incidence of diarrhea was also observed after administration of the water decoction, which indicated that restoration of the absorptive functions of the colon was markedly altered by the inflammatory process [26]. The effect of the H-*PHL* was the same as or better than that of a positive drug. Therefore, we believe that the *PHL* water decoction can have excellent anti-inflammatory effects on TNBS-induced intestinal inflammation. According to our results, we have shown that H-*PHL* had a protective effect on rats with TNBS-induced colon damage.

To further explore the underlying molecular mechanisms responsible for the therapeutic effects of *PHL*, biochemical determinations and immunohistochemistry were performed. In our study, the H-*PHL* water decoction reduced the activity of MPO, an enzyme present in neutrophils, catalyzing the formation of potent cytotoxic oxidants such as hypochlorous acid upon reaction with H_2_O_2_ and Cl^−^. Moreover, the MPO activity reflected the degree of neutrophil infiltration in intestinal inflammatory processes [7, 27]. As a result, MPO activity is a good marker of neutrophil infiltration [28], which can increase the cellular activity once access has been gained to the inflamed areas of the colon and tissue destruction is promoted by the release of reactive oxygen metabolites and proteolytic enzymes, and has been regarded as one of the indicators of pathogenesis in IBD in some previous studies [29]. Thus, a reduction in MPO activity, a reliable marker of reduced intestinal inflammation, in the animals treated with the water decoction may contribute to less neutrophil infiltration, which could lead to less colon tissue injury during intestinal inflammation, and therefore, it can also be interpreted as a sign of the anti-inflammatory effect of the *PHL* water decoction [30].

The recruitment and activation of neutrophils during acute inflammation contribute to the overproduction of reactive oxygen and nitrogen species overcoming the tissue antioxidant protective mechanisms, resulting in oxidative stress, which plays a significant role in the pathogenesis of IBD [31]. Excessive production of ROS in mucosal cells induces inflammatory and immune responses which can directly or indirectly cause damage to intestinal epithelial cells and, subsequently, disrupt the integrity of the intestinal mucosa barrier or initiate an inflammatory signaling cascade leading to severe impairment in experimental colitis [32]. Therefore, this study also investigated oxidative stress markers such as the glutathione content.

GSH, an endogenous antioxidant protecting cells from oxidative damage, allows the sulphhydryl groups (–SH) of proteins to maintain a relatively low level under physiological conditions and prevents them from reacting with free radicals [33], which is essential for controlling the redox state of cells [34]. Concentrations of endogenous antioxidants, such as GSH, are reduced significantly in patients with inflammatory bowel disease and in experimental models of colitis [35, 36], and it has also been reported that glutathione supplementation can reduce the severity of oxidative damage in TNBS colitis [37, 38]. This was indeed the case in our study, and a restoration of colonic GSH levels was observed after H-*PHL* treatment, which suggested that H-*PHL* had protective effects in intestinal inflammation probably due to its free radical scavenging and antioxidant properties. This may be an important and underlying mechanism for the protective effect of the *PHL* water decoction against intestinal inflammation.

In addition, it is generally accepted that the expression of inflammatory proteins, including cyclooxygenase- (Cox-) 2 and inducible nitric oxide synthase (iNOS), plays a pivotal role in modulating inflammation [39]. Production of Cox-2 and the activity of iNOS produce excessive inflammatory mediators which may aggravate the severity of intestinal damage [40]. Notably, increased activity of iNOS led to excessive synthesis of micromolar quantities of NO well above physiological levels [41, 42], which could be responsible for the pathological features of ulcerative colitis including interference with direct cytotoxicity, injury to gut epithelial cells, activation of neutrophils, and vasodilatation [43]. In addition, it can also inhibit key enzymes in the mitochondrial election transport chain and react with superoxide forming the highly toxic peroxynitrite radical, which can reversibly increase the activity of iNOS by activating NF-*κ*B, leading to a vicious effect cycle [44]. In the present study, we found that the production of Cox-2 and the activity of the iNOS level in the colon of rats with TNBS-induced intestinal inflammation increased markedly, while water decoction treatment reduced these levels. Thus, we believe that H-*PHL* has a protective effect on intestinal injury by significantly downregulating the production of Cox-2 and the activity of iNOS. This is in agreement with previous studies which showed that treatment with Cox-2 inhibitors could reduce the risk of gastrointestinal ulcer complications [45]. The severity of colonic inflammation was also reflected by iNOS and Cox-2, and the severity of colonic inflammation induced by TNBS was ameliorated by treating with SAZ and H-*PHL*, and the effect of H-*PHL* was slightly superior to that of SAZ.

H-*PHL* exhibits a relatively high suppression of the levels of cytokines, such as IL-1*β* and TNF-*α*, which are increased in inflamed tissue, including the mucosa of IBD lesions [46, 47]. The overproduction of cytokines is a key step in the inflammatory process and inflammatory cell infiltration [48], and these cytokines can not only increase the adherence and recruitment of leucocytes in the microvessels in chronic disease through upregulation of adhesion molecules to vascular endothelial cells [49] but also increase levels of tissue-specific and inflammatory chemokines and increase leucocyte migration [46]. Among these inflammatory cytokines, TNF-*α*, regarded as the most prominent “first-line” cytokine, plays a crucial role particularly in the development of intestinal inflammation, and it is synthesized and secreted by macrophages, lymphocytes, and polymorphonuclear neutrophils [50]. TNF-*α* is also implicated in the production of reactive oxygen species and oxidative stress-responsive genes which increase and prolong inflammation. Moreover, it has overlapping and synergic activities to induce the production of nuclear factor kappa B (NF-*κ*B) and other cytokines [8]. This offers a reasonable explanation of the effect of H-*PHL* reducing the inflammatory response through inhibition of proinflammatory cytokines in intestinal injury resulting from TNBS-induced intestinal inflammation in rats. One possible mechanism may involve downregulation of the inflammatory response by inhibiting the synthesis and release of proinflammatory mediators.

In the Western blot experiment, we found that the NF-*κ*B signal pathway was activated in the rats with TNBS-induced intestinal inflammation but blocked after treatment with H-*PHL* by detection of the NF-*κ*B subunit p65. NF-*κ*B proteins are a major family of transcription factors, composed of subunits of the Rel family, p50 and p65 [42], which play a key role in the regulation of proinflammatory gene transcription such as IL-1*β*, IL-6, TNF-*α*, Cox-2, iNOS, and adhesion molecules in the process of intestinal inflammation [51, 52]. Some investigations have shown that the inhibition of NF-*κ*B activity may reduce the severity of inflammatory diseases [53]. Furthermore, as NF-*κ*B is the final common pathway or rate-limiting step in the inflammatory cascade, if the therapy interferes with the NF-*κ*B pathway (the upstream target) in the cascade of inflammation, it will block the expression of multiple proinflammatory genes simultaneously, which might be more effective than suppressing individual factors such as TNF-*α*, IL-1*β*, and IL-6 in the treatment of intestinal inflammation [1]. Thus, in the present study, the anti-inflammatory effect of H-*PHL* may be to downregulate NF-*κ*B activity in TNBS-induced intestinal inflammation in rats and blockade of NF-*κ*B signal transduction pathways may be one of the major anti-inflammatory mechanisms of action of the water decoction.

In summary, all the findings above confirmed that the aqueous extract of *PHL* has excellent anti-inflammatory effects on TNBS-induced intestinal inflammation through modulation of the oxidant/antioxidant balance in colonic tissue. Finally, it is worth mentioning that this research was the first to demonstrate the effect of an aqueous extract of *Polygonum hydropiper* L. on TNBS-induced intestinal inflammation in rats and to identify the potential underlying protective mechanism of *Polygonum hydropiper* L. in TNBS-induced intestinal inflammation. Our research provided an initial theoretical and experimental basis for the wider use of an aqueous extract of *Polygonum hydropiper* L. in clinical situations or in the treatment of IBD. Our study results demonstrated that the aqueous extract of *Polygonum hydropiper* L., as a natural antioxidant and inflammatory inhibitor, was both effective and inexpensive, suggesting that *PHL* is a promising treatment for intestinal inflammation.

## Abbreviations

TNBS: 2,4,6-Trinitrobenzenesulphonic acid
CMC-Na: Sodium carboxymethyl cellulose
H-*PHL*: High dose of *Polygonum hydropiper* L.
IBD: Inflammatory bowel disease
L-*PHL*: Low dose of *Polygonum hydropiper* L.
M-*PHL*: Medium dose of *Polygonum hydropiper* L.
NII: Nonintestinal inflammation
*PHL*: *Polygonum hydropiper* L.
SD: Sprague Dawley
SPF: Specific pathogen-free
SAZ: Sulphasalazine
UC: Ulcerative colitis.
